# Highlight report: New methods for quantification of bile canalicular dynamics

**DOI:** 10.17179/excli2015-763

**Published:** 2015-12-21

**Authors:** Ahmed Ghallab

**Affiliations:** 1 Forensic Medicine and Toxicology Department, Faculty of Veterinary Medicine, South Valley University, Qena, Egypt

## ⁯

Cholestasis represents one of the main causes of hepatotoxicity. It is defined by impaired bile flux from hepatocytes to the gall bladder. Cholestasis can be caused by inhibition of the transport of bile acids from hepatocytes into bile canaliculi. Moreover, physical obstructions or impaired flux in the canaliculi or bile ducts may be responsible. Since decades cultivated hepatocytes have been used to study the cholestatic properties of chemicals (Dunn et al., 1989[7]). Tests in this field are based on the release and subsequent analysis of the canalicular content (Liu et al., 1999[22][23][24]; Bi et al., 2006[1]; Kostrubsky et al., 2003[20]). However, relatively little is known whether compromised bile canalicular dynamics, the rhythmic contractions of bile canaliculi, play a role in cholestasis. Therefore, Raymond Reif and colleagues have established an *in vitro* system that based on time-lapse microscopy allows the quantification of bile canalicular dynamics (Reif et al., 2015[29]). This technique measures the repetitive swelling and collapsing of bile canaliculi. This period typically lasts for approximately 30 h after plating of mouse hepatocytes. Afterwards, the luminal diameter of the bile canaliculi becomes narrower and more constant (Reif et al., 2015[29]). In this state it resembles morphology and dynamics of bile canalicular structures *in vivo* (Reif et al., 2015[29]). An interesting observation is that exposure to dexamethasone/insulin increased both, the amplitude of the initial contractions during the first 30 h and also the diameter of the mature canaliculi between 30 and 75 h after plating of the hepatocytes. In conclusion, Reif and colleagues have established a relatively easy to handle technique to quantify bile canalicular dynamics and they have shown that this parameter can be influenced by chemicals. However, whether compromised bile canalicular dynamics play a key role in pathogenesis of cholestasis still remains to be studied in future. 

Prediction of hepatotoxicity represents a cutting edge topic in toxicology (Ghallab et al., 2015[12]; Godoy et al., 2009[13], 2013[14], 2015[15]; Kim et al., 2015[19]; Hengstler et al., 2014[18]; Drasdo et al., 2014[4][5]; Frey et al., 2014[8]; Mazzanti et al., 2015[25]; Tolosa et al., 2015[35]; Chen et al., 2014[3]; Vitins et al., 2014[37]; Liu et al., 2014[21]). An intensively studied field are *in vitro* systems for hepatotoxicity, because they may improve the throughput and avoid difficulties due to interspecies extrapolation, because human cells can be used (Schyschka et al., 2013[31]; Messner et al., 2013[26]; Wobus and Löser, 2011[38]; O'Brien et al., 2006[27]). However, because of their limitations, *in vitro* systems have also been critically discussed (Reif, 2014[28]; Ghallab, 2013[10], 2014[11][9]; Stöber, 2014[34]; Hammad, 2013[17]). Many *in vitro* systems in the field of hepatotoxicity focus on alterations in gene expression caused by the test compounds (Shinde et al., 2015[32][33]; Campos et al., 2014[2]; Rodrigues et al., 2013[30]; Driessen et al., 2013[6]; Tolosa et al., 2013[36]; Grinberg et al., 2014[16]). Other endpoints, such as bile canalicular dynamics have been studied only rarely, possibly due to technical challenges. Therefore, it is fortunate that techniques for analysis of bile canalicular dynamics are now available. However, if - and if yes to which degree - compromised motility of bile canaliculi contributes to hepatotoxicity still has to be elucidated. 
